# Ultrasound phantom with solids mimicking cancerous tissue for needle breast biopsy

**DOI:** 10.55730/1300-0527.3454

**Published:** 2022-08-10

**Authors:** Işık İpek AVCI YAYLA, Melis BİLAL, Artür SALMASLIOĞLU, Erol ERÇAĞ, Esma SEZER, Belkıs USTAMEHMETOĞLU

**Affiliations:** 1 Department of Chemistry, Faculty of Science and Letters, İstanbul Technical University, İstanbul; 2 Department of Radiology, Facultyof Medical, İstanbul University, İstanbul; 3 Department of Chemistry, Faculty of Science, Namık Kemal University, Tekirdağ

**Keywords:** Self-healing polyacrylamide hydrogels, psyllium, cancerous tissue containing breast phantom, ultrasonographic biopsy

## Abstract

This study aimed at synthesizing hydrogels to simulate opaque breast tissue (BT) and coloured cancerous tissues (CT) at different densities of the designed phantom to improve the biopsy-related skills along with ultrasonography. Both tissues are tear-resistant and therefore, the phantom can be trained multiple times in order to lower the price and improve the eye-hand coordination of users. For this purpose, self-healing (SH) polyacrylamide (PAAm) hydrogels (SH hydrogel) obtained by free-radical polymerization of AAm, in the presence of chemical cross-linker, BAAm, physical cross-linker stearyl methacrylate, C18, and ammonium persulfate APS as initiator were used in the design of phantoms. Psyllium was added to the BT to differentiate density and obtain human skin color and it could be distinguished from the CT which was also colored with methyl violet. BT and CTs were characterized with FTIR spectroscopy, mechanical, swelling, and refractive index measurements. Designing phantoms from BT and CT were characterized by ultrasonography, mechanical tests, observation of needle track after biopsy, and stabilization tests to follow the self-healing behaviours of tissues with time. As a result of this study, self-healing, low-cost, and suitable for multi-usage ultrasonographic phantom for needle breast biopsy was designed and cancerous tissue was successfully detected.

## 1. Introduction

Breast cancer is the most frequent type of cancer among women in the world and constitutes 30% of all types of cancer observed in women [1]. According to the data in the year 2009, one of every four women has breast cancer and it is 24.1% of all cancer types. Although there are improvements in the medical sciences, development of early detection methods, and increased sensitiveness of the public, breast cancer continues to threaten life. Another step to decrease this threat is to increase the number and experience of specialized personnel and also of research opportunities. Therefore, this study aims to synthesize and characterize hydrogels and, design a phantom used to determine cancerous tissues by biopsy along with ultrasonography.

Training phantoms, simulating every part of the human body, are developed by diagnostic, X-ray, multiple modeling, mammography, radiation therapy, and ultrasound methods [2] which developed since 1960 [3]. Training phantoms are released by global firms such as Kyoto Kagaku (ABDFAN^®^-about 9500$) and CIRS (Zerdine^®^-about 3000$) [4]. Repeated use of phantoms caused needle tracks, which were a problem, and solved with patented material, zerdine. However, commercial phantoms are expensive and hard to prepare, therefore there are a lot of studies to design alternative noncommercial, low-cost phantoms. For the materials which will be used to simulate breast tissue (BT), the average velocity of sound should be 1540 m/s for better ultrasonographic performances [5]. In this study, the acoustic properties of PAAm hydrogels as a tissue material were investigated by designing a test phantom for ultrasonography.

The most popular materials reported in the literature for phantom design, are agar [6], graphite [7], polyurethane foam [8], magnesium silicate gels [9], SiC powder [10], natural gelatin [11], polyacrylamide gel [12], polyurethane [13], polyvinyl alcohol [14], thickened milk [15], urethane rubber [15], and cornstarch in gelatin suspension [16]. In another study, it is reported a phantom that was made by using gelatin and psyllium [17]. In another study, they used grapes or gloves filled with water as tumorous tissue and solid particles like macaroni, carrot pieces, or olives. Xu et al. [18] designed a phantom as tendon placed in swine muscle and biopsy showed less needle marks than gelatin-based phantoms after multiple biopsies. Silver et al. prepared another phantom by using macaroni and carrot particles to simulate targeted mass in bowels filled with agar [6]. This phantom is generally suitable for one week long and its cost was reported to be 20$. Osmer designed a phantom using macaroni and grapes as targeted mass [19].

In earlier works, psyllium was reported to use in pharmostatic, cosmetic, and alternative green ingredients in the food industry [20] as well as having ultrasonographically similar to the consistency of human soft tissue. Thus, in this study, psyllium was used as an ingredient since it is compatible with hydrogel, protecting its flexible structure, representing the soft tissue, and therefore enhancing the acoustic properties and appearance, easily accessible and cost-effective.

Synthetic hydrogels resemble biological tissues, and therefore, they are important materials in drug delivery and tissue engineering [21]. By inspiring from natural healing processes [22–24], synthetic hydrogels, which are healing themselves with the action of temperature [25], pH [26], or automatically [27], are designed. Healing requires bonds that are reversibly broken, thus preventing the main skeleton from collapsing. SH hydrogels were reported by creating strong hydrophobic interactions among hydrophilic polymers [28–31]. Some kinds of SH hydrogels have been prepared by a hybrid approach formed by a combination of physical and covalent networks [32]. This kind of hydrogels can be achieved by the copolymerization of hydrophilic monomers like AAm with the hydrophobes like stearyl methacrylate (C18) in an aqueous solution of sodium dodecylsulfate (SDS) in the presence of sodium chloride (NaCl) [28] and this type of reactions provide SH properties without the need of any heating or stimulus [27, 29–31].

Our goal was a design of a training phantom with an appearance similar to human soft tissue under ultrasound scanning, robust enough to withstand the pressure from ultrasound probe scanning. For this purpose, in light of information in the literature, we reported the preparation of SH hydrogels simulating BT, starting from AAm as a monomer, BAAm as a chemical cross-linking agent, and C18 as a physical cross-linking agent. Another hydrogel that mimics cancer tissue (CT) was obtained with the same method, by changing the amount of crosslinker and coloring the hydrogel. During ultrasonographic imaging, in order to have suitable contrast with BT and CT, psyllium was added into BT stimulating gel. Thanks to the self-healing property of hydrogel, the fast healing of needle tracks that occurs during biopsy make phantom suitable for multiple uses and an alternative to costly phantoms prepared from patented materials.

## 2. Experimental

### 2.1. Materials

Acrylamide (AAm), N,N-methylene bisacrylamide (BAAm), ammonium persulfate (APS), tetramethylethylene diamine (TEMED), dodecyl benzenesulfonic acid sodium salt (DBSA), sodium chloride (NaCl), stearylmethacrylate (C18), and psyllium (Solgar), Sipex trademarked RTV 1015 coded silicon and its catalyst, and toluene were used as received.

### 2.2. Characterization

#### 2.2.1. Equipment

For the preparation of hydrogels, mechanical and magnetic stirrer, Sonoplus HD 2070 ultrasonic homogenizer were used. For the characterization of gels, Perkin Elmer Spectrum One Fourier Transform infrared spectrometer (FTIR), Abbe refractometer for refractive index measurement, mechanical test via Zwick Z10 TS model device. For the storage of hydrogels, TNTONLIFE trademarked house-type vacuum device was used. The characterization of phantoms was performed using Siemens Sonoline Antares ultrasound device (Siemens, USA), using a high frequency, wide-band, linear probe (VFX 13-5).

### 2.3. Preparation of PAAm hydrogels

#### 2.3.1. Self-Healing PAAm hydrogels (SH hydrogel)

The BT simulating SH hydrogel was prepared via free-radical polymerization as suggested in the literature ^[32]^ (Sheme 1). For the synthesis of SH hydrogel which simulates the BT, first 0.24 M DBSA which was used as a surfactant was dissolved in a specific volume of water in order to have ultrasonographically similar consistency to human soft tissue since psyllium is compatible with hydrogel and has acoustic properties, 1.58 g psyllium was added to 0.5 M NaCl solution and stirred via mechanical stirrer until it was homogeneously dissolved. Then, C18 as a physical crosslinking agent was dissolved in this solution at 35 °C and stirred at a rate of 1000 rpm for 2 h. 1.29 M AAm and different concentrations of BAAm as a chemical crosslinking agent were added to this solution and stirred at a rate of 750 rpm for 30 min. Different SH hydrogels, simulating the BT were obtained in the presence and absence of psyllium by using different concentrations of BAAm and are given in Table 1.

Then 0.0035M APS as the initiator and 0.02 M TEMED as a reagent to speed up, were added to this solution and stirred for 2 min. After 10 min, the gelation was completed.

Throughout the synthesis of SH hydrogels, simulating the CT, 1.29 M AAm, 0.0066 M BAAm, 0.24 M DBSAS, 0.5 M NaCl, 0.0035 M APS, 0.025 M C18, and 0.02 M TEMED were used. Since the CT cannot be seen with the naked eye during the biopsy, it can only be detected ultrasonically, it is important that the piece taken is colored in order to be able to recognize whether it is from the CT. For the colorization of the CTs, 0.05 mL of 0.068M methyl violet solution was added. BAAm and C18 concentrations were changed and the resulting CTs were placed in BTs and different phantoms were obtained (Table 2). By the comparison of the ultrasonic images of these phantoms, optimum conditions were determined. BT_2_ and CT_2_, which are the hydrogels satisfying the optimum conditions, were kept on getting used for the rest of the experiments.

**Figure 1:**
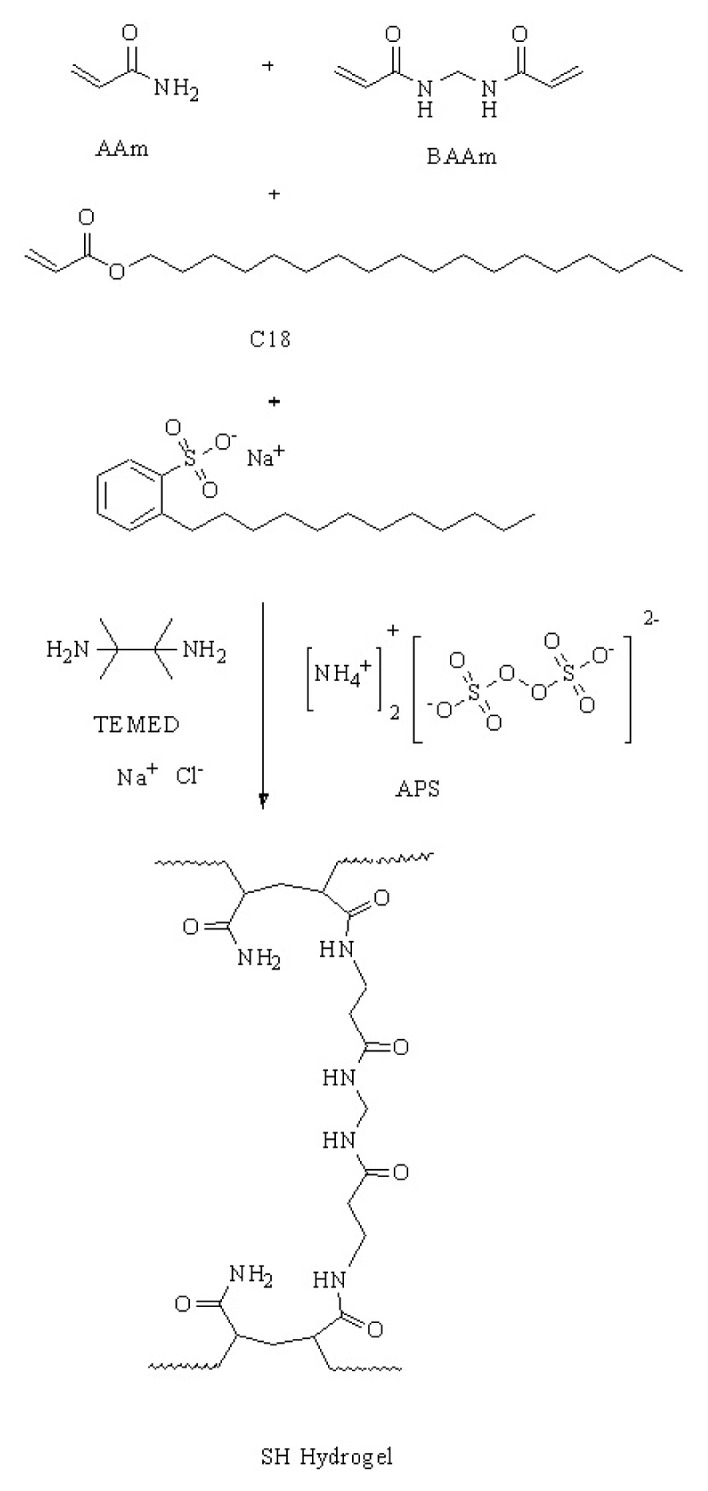
Mechanism of synthesis of SH hydrogels.

Mechanism of synthesis of SH hydrogels beginning with AAm, BAAm, C18, DBSA, TEMED, and APS is given in Figure 1.

#### 2.3.2. Nonself-healing PAAm hydrogels (NSH hydrogel)

For comparison, NSH hydrogels simulating the BT were also synthesized by free radical polymerization of AAm by using BAAm as the crosslinker and APS as the initiator ^[33]^. For the synthesis of NSH hydrogels, 0.703 M AAm, 0.0086 M BAAm, and 1.58 g psyllium-containing solution were prepared. Then 0.00175 M APS and 0.016 M TEMED were added and approximately after 45 min, gelation was observed. Similarly, for the synthesis of violet NSH hydrogels simulating the CT, 0.703 M AAm, 0.0129 M BAAm, 0.00175 M APS, 0.134 M TEMED, and 0.05 mL of 0.068M methyl violet solution were used. Gelation was completed approximately after 8 min.

#### 2.3.3. The phantom preparation

**Figure 2:**
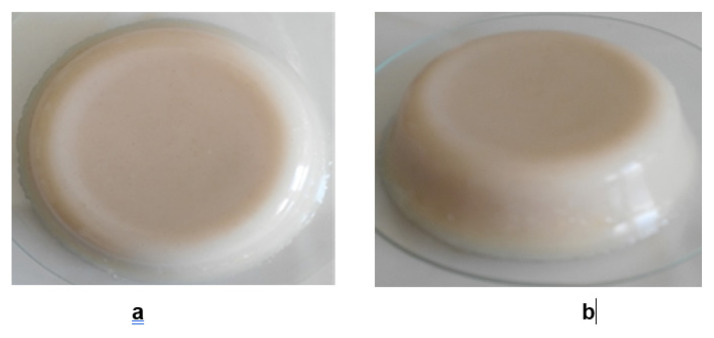
Images of SH-P **a)** top and **b)** side views.

When designing a self-healing phantom (SH-P), first the CT_2_ was synthesized. The reaction mixture was prepared for the BT_2_ and poured into a proper container for the final gelation. Towards the midst of the total reaction time, as soon as the gelation begins, the CT_2_ was placed in the middle of the BT_2_. For this reason, a certain reaction time is necessary for the stiffness of the gel. In order to hang the CT in the middle of the BT and not be visible, it must be placed in the first half of the reaction mixture that will form the BT, as soon as the reaction has progressed to a certain percentage, and the second half of the reaction mixture that will form the BT must be added after the CT is placed. A second important aspect of this time being critical is that when the time is exceeded, a line will form between the two BT mixtures interface after gelation had completed, which is undesirable in the ultrasonic view. Therefore, CT was placed inside the BT without getting sunk to the bottom or touching the sides, and this whole mixture was kept in this situation until the gelation is completed. Top and side views of completed SH-P are given in Figure 2.

On the other hand, for comparison, nonself-healing phantom (NSH-P) was prepared from NSH hydrogel for simulating BT and NSH hydrogel for simulating CT.

#### 2.3.4. The coating of the phantom with a PDMS elastomer

**Figure 3:**
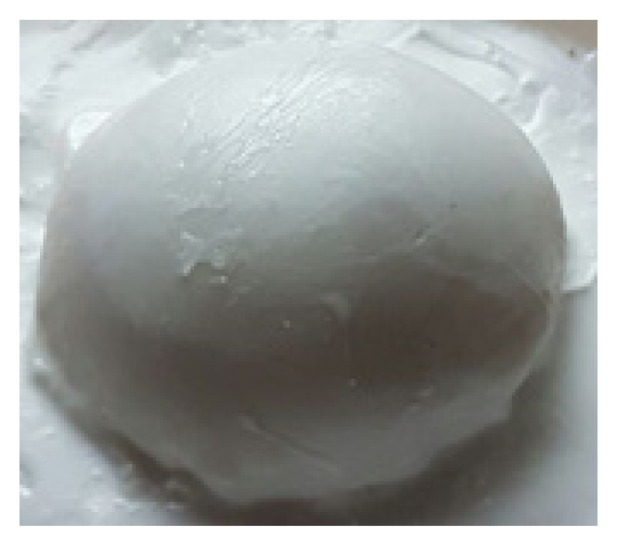
The top view of PDMS coated SH-P.

The phantoms are made from hydrogels and they easily lost their water. In order to protect them, their surface was covered with commercial PDMS elastomer. For this purpose, a molt was prepared from a PDMS elastomer starting from silicone oil by using 50% toluene, 45% silicone oil, 5% catalyst with respect to the total weight. By pouring this mixture into a breast shape mold, a homogeneous, opaque PDMS cover was obtained which takes approximately 15 min. The PDMS cover was taken out carefully by checking whether if there is any tear. The synthesis of PAAm hydrogels took place in this mold. The uncovered bottom side of the phantom was then covered with the same PDMS and the phantom was completely closed and has been protected from air and makes it reusable several times (Figure 3).

#### 2.3.5. Storing the phantom in vacuum

The amount of air intake of the phantoms could be reduced by coating the phantom with PDMS and the usage time could be extended. In order to prolong this period, the phantom was vacuumed and stored in a special vacuum bag with a household vacuum device with a power of 100 W.

## 3. Results and discussion

The SH hydrogels which were synthesized for BT and CTs were characterized by refractive index, swelling, FT-IR, measurements and mechanical tests before designing phantom,

### 3.1. Refractive index measurements

When the refractive index values of BT_2_ and CT_2_, violet CT were compared with the water’s refractive index, BT_2_ and CT_2_ seemed to have higher values due to having a higher density as a result of chemical and physical crosslinks (Table 3). Since BT_2_ contains psyllium, it is expected to have a higher refractive index value than CT_2_.

As a result, self-healing gels with a high crosslink degree and also containing psyllium have a higher refractive index. Although the refractive index difference between BT_2_ and CT_2_ is small, it is expected to make a difference in ultrasound imaging.

### 3.2. Swelling measurements

Swelling amounts of violet CT, BT_2_ and CT_2_ hydrogels were measured at different temperatures and are summarized in Table 4. Since BT_2_ and CT_2_ possess physical and chemical crosslinks, they are denser than NSH hydrogel. As the temperature was increased in both cases, the swelling amounts were also increased. At 25 °C, less dense CT_2_’s swelling amount was observed to have a higher value compared to denser BT_2_. This is thought to be caused by the psyllium which might be blocked some percentage of water uptake of SH-PAAm hydrogel. The swelling of psyllium was affected by temperature and become more efficient at 75 °C. Therefore, BT_2_ has a higher water adsorption value than CT_2_.

As a result, the different water uptake properties of BT_2_ and CT_2_ are expected to improve contrast, which leads to better ultrasonic imaging.

### 3.3. FT-IR measurements

**Figure 4:**
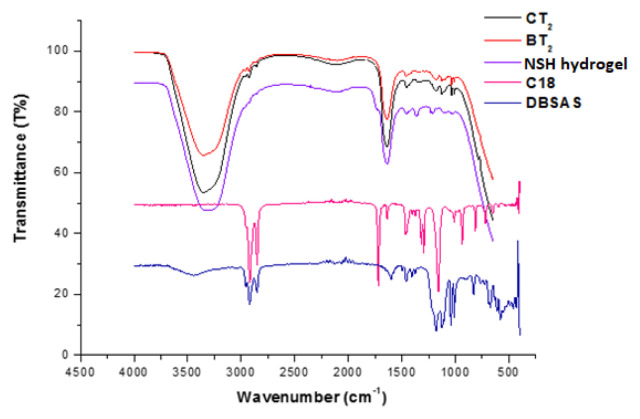
FT-IR spectra of CT_2_, BT_2_, NSH-hydrogel, C18, and DBSA.

In order to examine structural differences, FT-IR spectra of CT_2_, BT_2_, NSH hydrogel, C18, and DBSAS were obtained (Figure 4). The wavelength and bond vibrations of their characteristic peaks are summarized in Table 5. In the FTIR spectra of BT_2_ and CT_2_, the presence of characteristic peaks of PAAm supported the formation of PAAm. Methyl violet’s peak was not observed since it was used in quite low concentrations as compared to other ingredients.

As it can be seen the observation of the characteristic peaks of C18 and DBSAS around 700–1000 cm^−1^, 2800–3000 cm^−1^ respectively for BT_2_ and CT_2_ indicates that C18 and DBSAS were included to the structure (Figure 4). There was no significant difference between the FT-IR spectra of BT_2_ and CT_2_, while the same peaks were absent in the FTIR spectrum of NSH-hydrogel since C18 and DBSA were not used during the synthesis.

### 3.4. Mechanical tests

**Figure 5:**
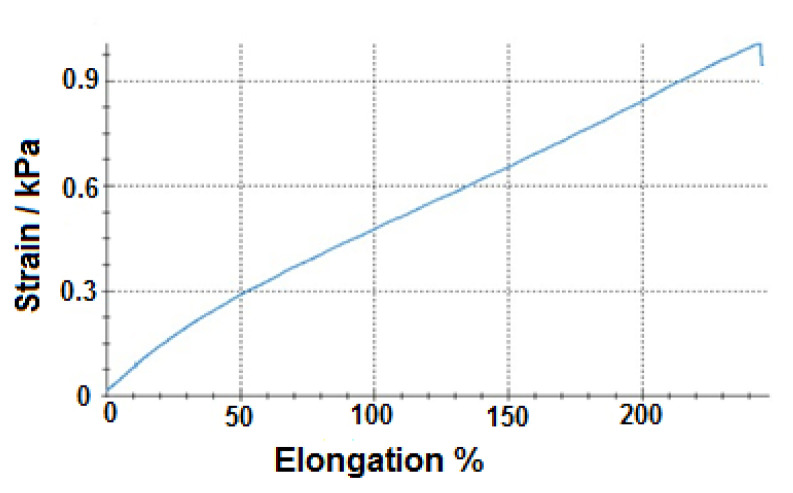
Strain-elongation graph of BT_2_.

In order to gain an idea about the mechanical properties of the hydrogels and phantoms, elongation and comparison tests were applied respectively. For elongation test, first, a bone-shaped standard samples were prepared from the BT_2_ and NSH hydrogel and elongation tests were applied. Figure 5 shows strain-elongation graph of BT_2_ and both results are compared in Table 6.

As it can be seen in Table 6, BT_2_ possesses threefold durability compared to NSH-hydrogel under the approximately same percentage elongation. This result exhibited that self-healing property has a positive outcome on the mechanical properties as it is expected and it was thought to be resulted by not only by chemical crosslinks but also with the help of physical crosslinks.

**Figure 6:**
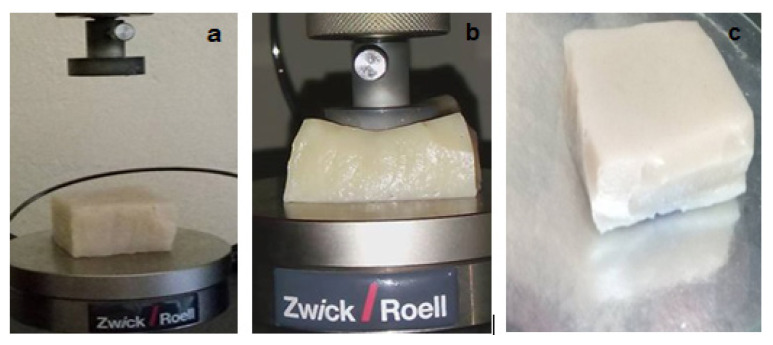
The images of SH-P **a)** before **b)** during, and **c)** after compression test.

**Figure 7:**
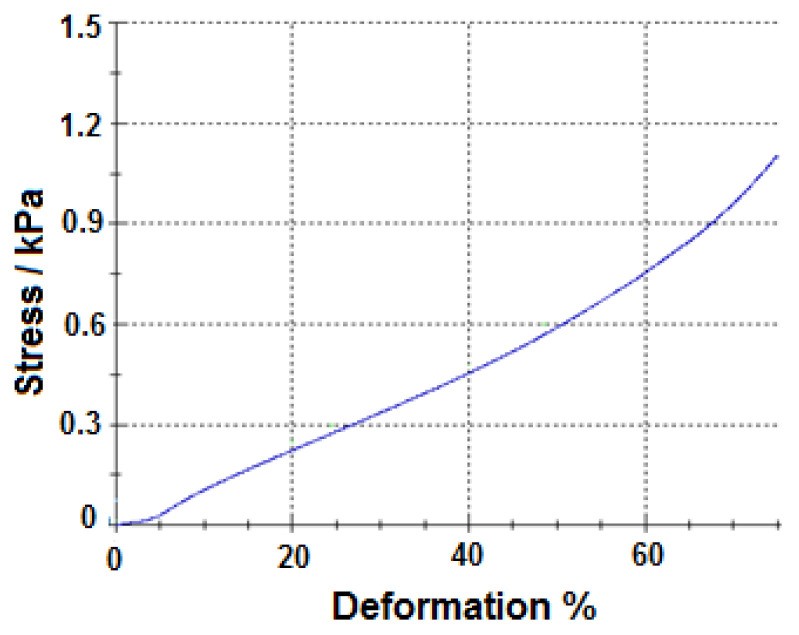
Stress–percentage deformation graph of SH-P.

The compression tests of the SH-P and NSH-P were performed on the samples with the sizes of 4.0 × 4.0 × 2.0 cm and compared. Stress values of both phantoms to obtain 65% deformation was recorded and the images obtained during that test are given in Figure 6 which there were no permanent changes after compression measurements. Stress–percentage deformation graph of SH-P is given in Figure 7. The stress values of NSH-P and SH-P were found as 2.10 kPa and 0.85 kPa respectively. As it can be seen, SH-P needed less pressure as compared to NSH-P. SH-P required 1.16 kPa under 75% deformation which was still lower than what was required for NSH-P. This result indicated that SH-P is more flexible than NSH-P and supports the tensile property test results. This flexibility of the structure is thought to be caused by the self-healing property.

### 3.5. Ultrasonographic measurements of the phantoms

**Figure 8:**
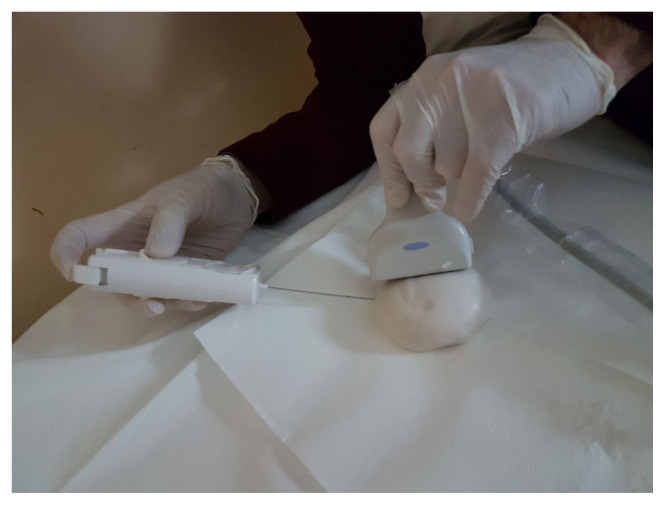
Ultrasound-guided needle biopsy of SH-P.

**Figure 9:**
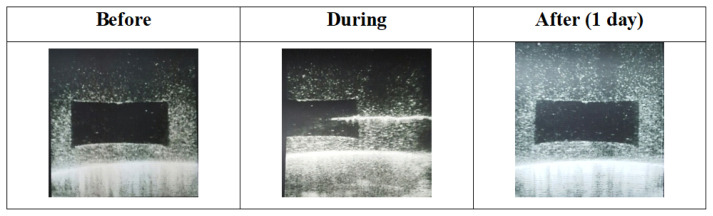
Ultrasonographic images of the SH-P just before, during, and after one day from biopsy.

**Figure 10:**
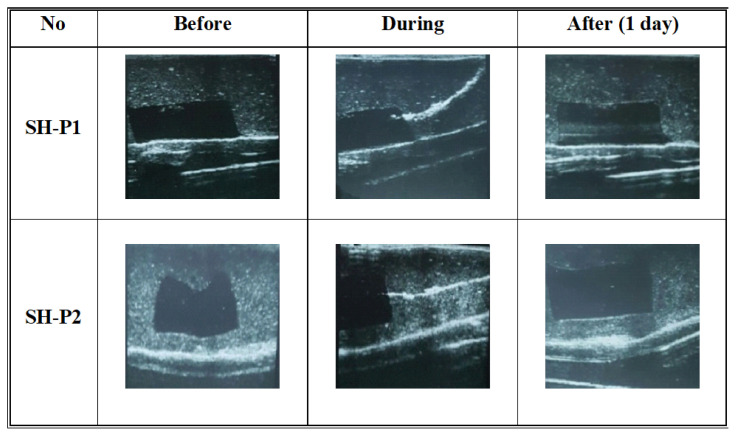
The effect of psyllium amount on the ultrasonographic images of the phantom just before, during, and after one day from biopsy.

**Figure 11:**
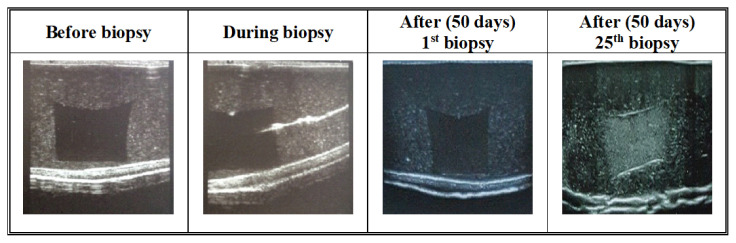
Ultrasonographic images of SH-P before, during and after first and fourth biopsy.

**Figure 12:**
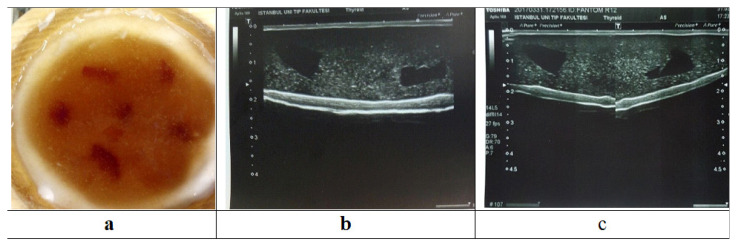
**a)** Image of top view of SH-P6 before coating with PDMS, **b)** ultrasonography images of 2 CTs in one frame and **c)** individual CT obtained from two different frames.

Designed ultrasound needle breast biopsy phantom with amorphous lesions, was used for ultrasound-guided needle biopsy training and ultrasonographic images were obtained before, during, and after biopsy (Figure 8). Results are given in Figures 9–12 and suggested that phantoms accurately mimic the ultrasonic characteristics of human breast tissues and ultrasound identification of CT was successfully performed.

Figure 9 showed ultrasonographic images of the designed SH-P. Since the phantoms were constructed of a self-healing formulation of SH hydrogels, the needle tracks healed after a day. As it can be seen needle tracks did not visible on PDMS cover.

In order to increase the resolution of the images of SH-P given in Figure 9, the phantom was prepared with an increased amount of psyllium in BT_2_. When the psyllium amount was increased to 2 g, the image became clear and the distinct recognition between the CT simulating hydrogels and BT simulating hydrogels could be obtained. This might be due to the changing the acoustic impedance and as a result, the contrast between the BT and CT would change. Figure 10 shows the ultrasonographic images of phantoms with the same psyllium (SH-P1) and the higher psyllium (SH-P2) amount. The rest of the studies was gone on with the latter one which had a clearer image.

In order to protect the water loss of phantom, it is stored in a special bag that is vacuumed via 100 W vacuum device during the biopsy intervals and when it is not used. The phantom was designed to allow multiple biopsy insertions with minimal needling tracking. In order to check this property, the biopsy was tried 4 times every day for 50 days Figure 11 shows the ultrasonographic images of the phantoms obtained just before, during, and after the biopsy. As it can be seen needle tracks usually disappear and CT may be biopsied multiple times.

Six CTs were randomly positioned in a phantom and the top view of the SH-P6 before coating with PDMS (Figure 12a) and ultrasonographic images are given in Figures 12b and c. Although all CTs could be seen individually, only 2 or 3 CTs could be visualized in a single frame because the width of the ultrasonography probe, which is approximately 6 cm, was not sufficient (even though it was wider than the normal probe used). Figure 12b is the multiple ultrasonography images of 2 CTs in one frame, and Figure 12c is the multiple ultrasonography images of individual CTs in two different frames.

As a result, it was observed that phantoms prepared with self-healing gels containing a certain amount of psyllium were more advantageous than NSH ones in terms of ultrasonic imaging and multiple uses.

## 4. Conclusion

According to the synthesis and the design presented in the study, breast and cancerous tissue simulating self-healing hydrogels was synthesized with two different densities and colors. Biopsy compatible, BT and CT mimicking phantoms were successfully imaged by ultrasonography and it benefits to improve hand-eye coordination, and build confidence and reduce patient anxiety of user during the training procedures. The desired properties of the phantom for biopsy training have been continued for at least 50 days, these phantoms did not show any damage and still exhibited self-healing to needle tracks in case it was stored under vacuum. When the costs of raw material for self-healing hydrogel synthesis are taken into consideration, the phantom obtained in this study costs 10 times less than the commercial Zerdine phantom.

In conclusion, new, cost-effective, easily attainable, containing solids that mimicked the texture of CT and can be biopsied, having healing properties for the needle tracks, and convenient phantoms which were suitable for training residents in ultrasound-guided needle biopsy were successfully designed.

## Figures and Tables

**Table 1:** Concentrations of BAAm, and psyllium for preparing SH hydrogel to simulate BT. [AAm] = 1.29 M, [DBSAS] = 0.24 M, [NaCl] = 0.5 M, [APS] = 0.0035 M, and [TEMED] = 0.02 M.

No	[BAAm,] M	Psyllium, g/100mL
**BT** **_1_**	0.0066	-
**BT** **_2_**	0.0066	1.58
**BT** **_3_**	0.0033	1.58

**Table 2:** Concentrations of C18, BAAm and psyllium for preparing SH hydrogel to simulate CTs. [AAm] = 1.29 M, [DBSAS] = 0.24 M, [NaCl] = 0.5 M, [APS] = 0.0035 M, [TEMED] = 0.02 M.

No	[C-18]	[BAAm] M	Psyllium g/100mL
**CT** **_1_**	0.500	0.0330	-
**CT** **_2_**	0.025	0.0066	-
**CT** **_3_**	0.025	0.0066	63.2

**Table 3:** Refractive index values of BT_2_, CT_2,_ NSH hydrogel, and water.

Substance	Refractive index
**BT** **_2_**	1.444
**CT** **_2_**	1.414
**NSH hyrogel**	1.352
**water**	1.333

**Table 4:** The water uptake percentage values of violet NSH-hydrogel, BT_2,_ and CT_2_ at two different temperatures.

Hydrogel	Water uptake %	
	25 °C	75 °C
**NSH**	77.97	91.43
**BT** **_2_**	125.73	385.98
**CT** **_2_**	232.39	324.82

**Table 5:** The wavenumbers and bond vibrations of the characteristic peaks of PAAm, NSH, CT_2_ and BT_2_, C-18 and DBSA.

Hydrogel	Wavenumber (cm^−1^)	Bond Vibration
**PAAm**	1500–1600	-C=O
	3200–3500	-N-H
	2950	-C-H
	3200–3500	-N-H
**NSH**	1500–1600	-C=O
	3200–3500	-N-H
**CT** **_2_**	1000–1060	-C-H(Alkene, Aromatic)
	1500–1600	-C=O
	2840–2960	-C-H(Alkan)
	3200–3500	-N-H
**BT** **_2_**	1000–1060	-C-H(Alkene, Aromatic)
	1500–1600	-C=O
	2840–2960	-C-H(Alkane)
	3200–3500	-N-H
**C18**	1000–1060	-C-H(Alkene)
	2840–2960	-C-H(Alkane)
**DBSAS**	1000–1060	-C-H(Aromatic)
	2840–2960	-C-H(Alkane)

**Table 6:** Strain and percentage elongation values of NSH hydrogel and BT_2_ at the breaking point.

Hydrogel	Strain, Pa	Elongation%
**NSH**	375	250
**BT** **_2_**	1060	244
